# Predicting tumor repopulation through the gene panel derived from radiation resistant colorectal cancer cells

**DOI:** 10.1186/s12967-023-04260-x

**Published:** 2023-06-16

**Authors:** Yanwei Song, Zheng Deng, Haoran Sun, Yucui Zhao, Ruyi Zhao, Jin Cheng, Qian Huang

**Affiliations:** 1 Cancer Center, Shanghai General Hospital, Shanghai Jiao Tong University School of Medicine, Shanghai, 201620 China; 2 Shanghai Key Laboratory of Pancreatic Diseases, Shanghai General Hospital, Shanghai Jiao Tong University School of Medicine, Shanghai, 201620 China

**Keywords:** Radiation resistance, Radiotherapy, Tumor repopulation, Colorectal cancer, Prognostic indicator

## Abstract

**Background:**

Tumor cells with the capability of radiation resistance can escape the fate of cell death after radiotherapy, serving as the main cause of treatment failure. Repopulation of tumors after radiotherapy is dominated by this group of residual cells, which greatly reduce the sensitivity of recurrent tumors to the therapy, resulting in poor clinical outcomes. Therefore, revealing the mechanism of radiation resistant cells participating in tumor repopulation is of vital importance for cancer patients to obtain a better prognosis.

**Methods:**

Co-expressed genes were searched by using genetic data of radiation resistant cells (from GEO database) and TCGA colorectal cancer. Univariate and multivariate Cox regression analysis were performed to define the most significant co-expressed genes for establishing prognostic indicator. Logistic analysis, WGCNA analysis, and other types of tumors were included to verify the predictive ability of the indicator. RT-qPCR was carried out to test expression level of key genes in colorectal cancer cell lines. Colongenic assay was utilized to test the radio-sensitivity and repopulation ability of key gene knockdown cells.

**Results:**

Prognostic indicator based on TCGA colorectal cancer patients containing four key radiation resistance genes (LGR5, KCNN4, TNS4, CENPH) was established. The indicator was shown to be significantly correlated with the prognosis of colorectal cancer patients undergoing radiotherapy, and also had an acceptable predictive effect in the other five types of cancer. RT-qPCR showed that expression level of key genes was basically consistent with the radiation resistance level of colorectal cancer cells. The clonogenic ability of all key gene knockdown cells decreased after radiation treatment compared with the control groups.

**Conclusions:**

Our data suggest that LGR5, KCNN4, TNS4 and CENPH are correlated with radiation sensitivity of colorectal cancer cells, and the indicator composed by them can reflect the prognosis of colorectal cancer patients undergoing radiation therapy. Our data provide an evidence of radiation resistant tumor cells involved in tumor repopulation, and give patients undergoing radiotherapy an approving prognostic indicator with regard to tumor progression.

**Supplementary Information:**

The online version contains supplementary material available at 10.1186/s12967-023-04260-x.

## Introduction

With the improvement of global average standard of living and the ability to diagnosis and treatment, colorectal cancer has gradually become the third most common tumor in past few decades [1, 2]. The risk factors may be related to obesity, lifestyle and genetic factor [1, 2]. Except for the improvement of diagnosis, the reason for the rise of incidence rate has not been fully understood [1, 2]. The treatment of colorectal cancer revolves around surgery. Depended on the stage of the disease, strategies may be combined with chemotherapy, radiotherapy, biological therapy, and immunotherapy [2]. As a key component of local treatment for colorectal cancer, radiotherapy, especially in neoadjuvant therapy of rectal cancer, aims to reduce local recurrence and improve survival for patients [2]. However, it is frequently observed in clinical practice that recurring lesions are more resistant to radiation than primary tumor and tend to proliferate faster. The reason may be that a group of special tumor cells were generated during the treatment process, which may exhibit resistance to radiation therapy and ultimately lead to treatment failure, tumor progression and poor prognosis in patients.

The above-mentioned phenomenon is a manifestation of tumor plasticity, which is probably related to cancer stem cells (CSCs), making tumor adapt to adverse environments including anti-cancer treatments induced stress, and being observed as therapeutic resistance and tumor progression eventually [3–5]. In other words, some tumor cells have undergone phenotypic evolution or natural selection (therapeutic-resistant subtypes that already existed in the early stages of tumorigenesis but were not dominant) under the pressure of treatment [3, 6]. There were also theories describing the treatment-resistant tumor cells as persistent cells or slow cycling cells [7–10]. These cells could escape treatment stress and enter a dormant phase during treatment to stop proliferation. When treatment was stopped, they re-entered the proliferative cycle and led to tumor recurrence, being a major component of treatment-resistant pool. Recently, there were also theories calling this process adaptive evolution, in which tumor cells increased mutations and responded to stress, and healthy subclones were screened [11]. This phenomenon has been described in therapy of several kinds of cancer, e.g., targeted therapy of lung cancer [12], hormone therapy of prostate cancer [13], immunotherapy and targeted therapy of melanoma [14]. These theoretical models all point to a group of residual drug-resistant cells, which become the source of therapeutic resistance and mediate tumor cells repopulation after anti-tumor therapy when sensitive cells died. Although radiotherapy is rarely used as an intervention in these theoretical models, the course of some colorectal cancer patients suggest that radiotherapy may also produce a group of radiation resistant cells which participate in tumor recurrence (tumor cell repopulation). Therefore, searching for biomarkers involved in radiotherapy resistance is of great significance for judging the treatment effect and tumor progression.

Given that some colorectal cancer patients have similar phenomena, from tumor regression after radiotherapy to recurrence (radiation tolerance), we focused our attention on radiation-resistance of colorectal cancer in this research. By studying the relationship between radiation-resistance and tumor repopulation, we explored some key genes that played a part in both of them, so as to predict the prognosis of colorectal cancer. In addition, we verified these genes in other types of tumors and in virto experiments to determine the reliability in predicting radiation-resistance and tumor progression.

## Materials and methods

### Project selection and data collection

In order to acquire the necessary data for this study, we used the public functional genomics data repository, Gene Expression Ominibus. By setting “colorectal cancer”, “radiation” and “resistance” as three keywords for obtaining desired project, we enrolled series GSE97543 as our mainly analyzing dataset eventually [15]. In this project, gene expression of radiation resistant colorectal cell line and its control group were described, and all GEO data were downloaded through package “GEOquery” of R software [16].

To further get gene expression and clinical data of patients, part of cases originated in TCGA (The Cancer Genome Atlas) program were involved. We collected gene expression data of colon, rectum and rectosigmoid junction adenocarcinoma (TCGA-COAD, TCGA-READ) cases, including 644 tumor samples and 51 normal samples to search target genes with GSE97543. Meanwhile, radiation treated TCGA-CO/READ patients (n = 35) were set as training set. Patients who received external radiotherapy as only adjuvant therapy in BRCA (breast carcinoma, n = 38), LUAD (lung adenocarcinoma, n = 36), CESC (cervical squamous cell carcinoma and endocervical adenocarcinoma, n = 20), HNSC (head and neck squamous cell carcinoma, n = 95) and ESCA (esophageal carcinoma, n = 17) were introduced to be validating parts. All TCGA data, mRNA expression and clinical details were manually downloaded from the website and organized by R software.

### Founding differential expression genes

Two R packages “limma” and “edgeR” were adopted to identify the differential expression genes (DEGs) in both databases [17, 18]. |Log2 fold change|≥ 1 and P. value ≤ 0.05 were set as the selection criterion, which defined the scope of DEGs in two databases for further analysis. And volcano plots were draw by R packages “limma” and “ggplot2”. A gene annotation and analysis resource website, “Metascape” (https://www.metascape.org/), was utilized to cluster gene function and to enrich pathways related to DEGs [19].

### Cox Regression analysis and construction of prognostic prediction mode

After acquiring 252 upregulated co-expression genes, which were candidates to construct radiation resistance gene panel, between GSE97543 and TCGA-CO/READ, we involved univariate Cox regression to pick out the most relevant genes with PFS (progression-free survival) of radiation treated colorectal patients with P. value < 0.06, which was set to prevent missing out clinically significant genes that closely approached the conventional significance level [20, 21].

Then, we developed a proportional hazards regression analysis (multivariate Cox model) by the following formula using the result of univariate Cox regression.

$$Risk\, score= {\sum }_{i=1}^{n}(Coefficienti\times xi)$$

In this equation, risk score is the mRNA expression of each key predictor genes (xi) multiplied by the coefficient (Coefficienti), the latter came from multivariate Cox regression analysis. Using the median value of risk score that calculated by the formula before, the 35 TCGA patients in the training set were divided into two groups, high risk group and low risk group. Based on this grouping routine, we observed the expression levels of key genes in the two groups and established a survival analysis between PFS and risk score. ROC (receiver operating character) curves were implemented to evaluate the predictive ability of radiation resistance gene panel using R package “survivalROC”, and the AUC (area under the curve) values were calculated to visualize inspection capabilities.

In order to test whether risk score was an independent prognostic factor, we incorporated the age, gender, stage, T, N, M status and risk score of 35 TCGA patients into the multivariate Cox regression model, so as to exclude confounding caused by other clinical features. Furthermore, R package “rms” was used to make a nomogram model, showing above clinical information.

### Testing the applicability of radiation resistance gene panel

Besides TCGA-CO/READ, we further chose patients who received radiotherapy as only adjuvant therapy from other TCGA projects to check validity of radiation resistance gene panel. Logistic regression models were built by R package “glmnet”, and different gene combination models were listed. Several best models and their AUC values of different cancer types were displayed respectively.

### WGCNA analysis

The R package, “WGCNA” (Weighted Gene Co-expression Network Analysis), was utilized to build a co-expression network targeting DEGs. This method aims to find co-expressed gene modules, and to explore the relationship between gene networks and the phenotype of interest, as well as the core genes in the network [22]. It is divided into two parts: expression cluster analysis and phenotypic correlation.

The specific method was that selecting patients who received radiotherapy in TCGA-COREAD, and incorporating the top ten thousand DEGs with the largest variance of these patients into the cluster analysis of gene expression. After clustering the samples and eliminating outliers, we determined the soft-thresholding power according to the algorithm, which was used to construct a scale-free co-expression network and determine gene modules. Each color represented a module, and each module contained genes with similar expression patterns. Dynamic tree cut analysis was developed to represent the classification of genes, and modules with high similarity were fused to construct merged dynamic clusters. In addition, different modules were established the correlation to two clinical traits, PFS and risk score, and modules that contained key radiation resistant genes were selected to carry out GO analysis.

### Cell culture

Immortalized human colorectal tumor cell lines (HCT 116 and HT-29) and 293 T cells used in this research were bought from cells bank of the Chinese Academy of Science (Shanghai, China). HCT 116, HT-29 and 293 T cells were seeded in completed DMEM medium (Wisent, China). 10% Fetal Bovine Serum (Wisent, China) and 1% Penicillin–Streptomycin (Wisent, China) were added to the completed DMEM medium. All cells were cultured in 5% CO_2_ at 37 ℃.

### Cell Irradiation and colony formation assay

An X-Ray generator (Faxitron, USA) for laboratory was used to treat cells. And the dose rate was 3 Gy/min. HCT 116 and HT-29 were seeded in six well culture dishes (100, 200, 1000, 2500 and 10,000 cells/well) and incubated eight hours (overnight) until adherent before irradiation. After adherent, cells were treated with various doses (0, 2, 4, 6, 8 Gy) of X-Ray respectively. All cells were fixed by paraformaldehyde of 4% fortnight later. And then, they were stained by crystal violet. This experiment was repeated three times independently. Any colony that contained fifty cells or more were counted and linear-quadratic model in Graphpad Prism 8 was employed to calculate the surviving fraction.

### Detection the expression level of mRNA by RT-qPCR

Total mRNA was extracted by using RNA Easy Fast Cell Kit (Tiangen, China). cDNA was reversely transcribed from mRNA by using the RT Kit. Q-PCR was conducted by using TB Green Kit. Relative mRNA level was calculated by the formula 2 − △△CT. The experiments were repeated three times. Both kits mentioned above were from Takara, Japan. Specific primers information is clarified in supplemental material. GAPDH, an internal reference gene, were used as a control for standardization.

### ShRNA transfected and knockdown cell lines constructed

All shRNA sequences were got from MERCK website. When the confluence of 293 T cells in the cell culture dish reached 80–90%, we mixed the target plasmid containing shRNA, tool plasmid psPAX2, pMD2.G and GFP fluorescent plasmid into the 400 μL Opti-MEM medium according to the protocol proportion, and then added the mixed system into 293 T cell culture medium. After 48 h, the virus solution from 293 T cells was obtained and added to HCT116 and HT29 cell culture medium with 10 μg/mL polybrene. After another 48 h, the virus group cells and control group cells were treated with 1.5 μg/mL purinomycin (the tool plasmid contained purinomycin-resistance gene to ensure that these cells successfully infected by the virus would not be killed). When the control cells were all killed by purinomycin, the surviving cells in virus group were thought to be successfully infected by the virus. Western Blot experiment was carried out to detect the expression level of genes.

### Western blot experiment

Cell protein was obtained by using cracking liquid to crack the cells. The obtained protein denatured by boiling, and SDS-PAGE gel was used for electrophoresis to separate proteins with different molecular weights. After electrophoresis, SDS-PAGE gel and PVDF (polyvinylidene fluoride) paper were used to constructed gel-matrix sandwich to transfer. After that, the PVDF paper was incubated successively with the first and second antibodies, and exposed in developer to obtain the image of the target band. These experiments were repeated three times independently. The blank controls were wild type HCT116 and HT29 cells without any treatment. The negative control referred to two types of cells transferred into empty plasmids.

### R Packages and statistic analysis

We conducted this research mainly based on R and R studio software. R packages employed include: “clusterProfiler”, “GEOquery”, “org.Hs.eg.db”, “HsAgilentDesign026652.db”, “R.utils”, “rjson”, “jsonlite”, “reshape2”, “ggfortify”, “limma”, “edgeR”, “GOplot”, “stringr”, “ggplot2”, “dplyr”, “pheatmap”, “glmnet”, “survival”, “survminer”, “rms”, “cowplot”, “WGCNA”, “ROCR”, “survivalROC”. The difference in progress-free survival in different groups of patients was analyzed using log-rank test in GraphPad Prism 8, and two tailed student’s t-test was used to compare mean values. * for P ≤ 0.05; ** for P ≤ 0.01; *** for P ≤ 0.001. Statistical significance was set at P ≤ 0.05 in most conditions, and specific principles are n.s for not significant.

## Results

### Sequencing data analysis of radiation resistant cells and TCGA patients

**Fig. 1:**
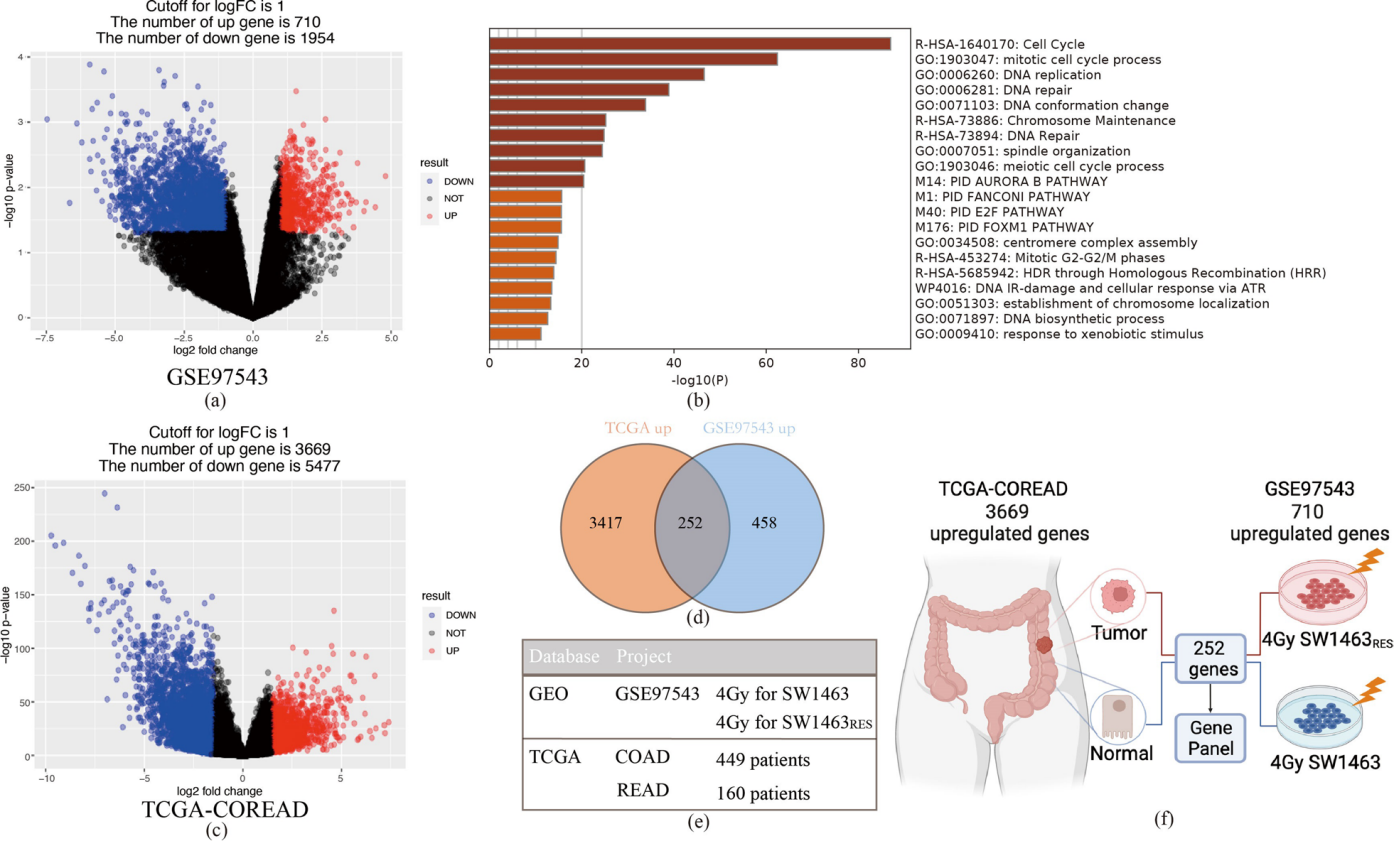
Genetic profiling and overall design to explore radiation resistance and tumor repopulation. **a** Volcano plot of radiation resistant cells vs. control cells in GSE97543. |Log2 fold change|≥ 1 and P.value ≤ 0.05 were set as cut-off values. Red dots were recognized as upregulated genes, while blue as down-regulated ones in DEGs analysis. **b** 710 upregulated genes were imported into Metascape website for pathway enrichment analysis. **c** Volcano plot of tumor vs. normal tissues in TCGA-CO/READ database. Cut-off values and colors were same as before. **d** Venn diagram showed the intersection of the upregulated genes in the TCGA and GEO databases. 252 co-upregulated genes were spotted. **e** Details of the data included in this study. Resistant rectal adenocarcinoma cells and control cells included in GEO database were both treated by 4 Gy radiation. TCGA database contained colon and rectal adenocarcinoma patients. **f** Visualization of the overall experimental design. 252 co-upregulated genes were used to screen key genes to form a gene panel

In order to study the genes that played a key role in radiotherapy resistance, we selected the dataset GSE97543 as the research object [15]. In this database, the radiation-resistant human rectal adenocarcinoma cell line SW1463 was established through repeated radiation exposure, named SW1463RES. Compared with control cells after 4 Gy radiation, SW1463RES presented 710 upregulated genes and 1954 down-regulated genes, when cut-off values were set as |Log2 fold change|≥ 1 and P. value ≤ 0.05 in DEGs analysis (Fig. 1a, Additional file 6: Table S1). In order to clarify the function of the obtained upregulated genes, we used the Metascape tool website to perform Gene Ontology (GO) analysis and pathway analysis on this group of genes, which primarily enriched in: (1) cell cycle related pathways (Cell cycle, Mitotic cell cycle process, Mitotic G2-G2/M phase, E2F pathway, FOXM1 pathway, DNA IR-damage and cellular response via ATR); (2) DNA replication and repair related pathways (DNA replication, DNA biosynthetic process, DNA repair, Fanconi pathway and Homologous recombination repair); (3) stress response pathways (response to xenobiotic stimulus) (Fig. 1b). According to the results of enrichment analysis, radiation resistant SW146RES cells exhibited stronger DNA damage repair capabilities than control cells, which were in accordance with previous views [23, 24]. Because these genes that represented radio-resistance may promote tumor to recover from the stress state caused by X-Ray beam more quickly, they were inclined to lead tumor repopulation after radiation. Therefore, we further conducted a DEGs analysis, tumor vs. normal tissue, in the TCGA-CO/READ databases, and acquired 3669 upregulated genes while 5477 down-regulated genes were spotted (Fig. 1c, Additional file 7: Table S2). By using the Venn plot to intersect the upregulated genes of two groups, we finally found 252 co-upregulated genes (Fig. 1d), and numerous similar pathways were enriched in cell cycle, DNA replication and repair (Additional file 1: Figure S1). The specific items we selected in the TCGA and GEO databases were listed in Fig. 1e, and the detailed process of how we used these genes expression data to build our practice was described through a visual flow chart (Fig. 1f). These 252 co-upregulated genes were considered as candidates for constructing the radiation-resistance gene panel.

### Screening key radiation-resistance genes related to clinical outcomes in colorectal cancer

**Fig. 2:**
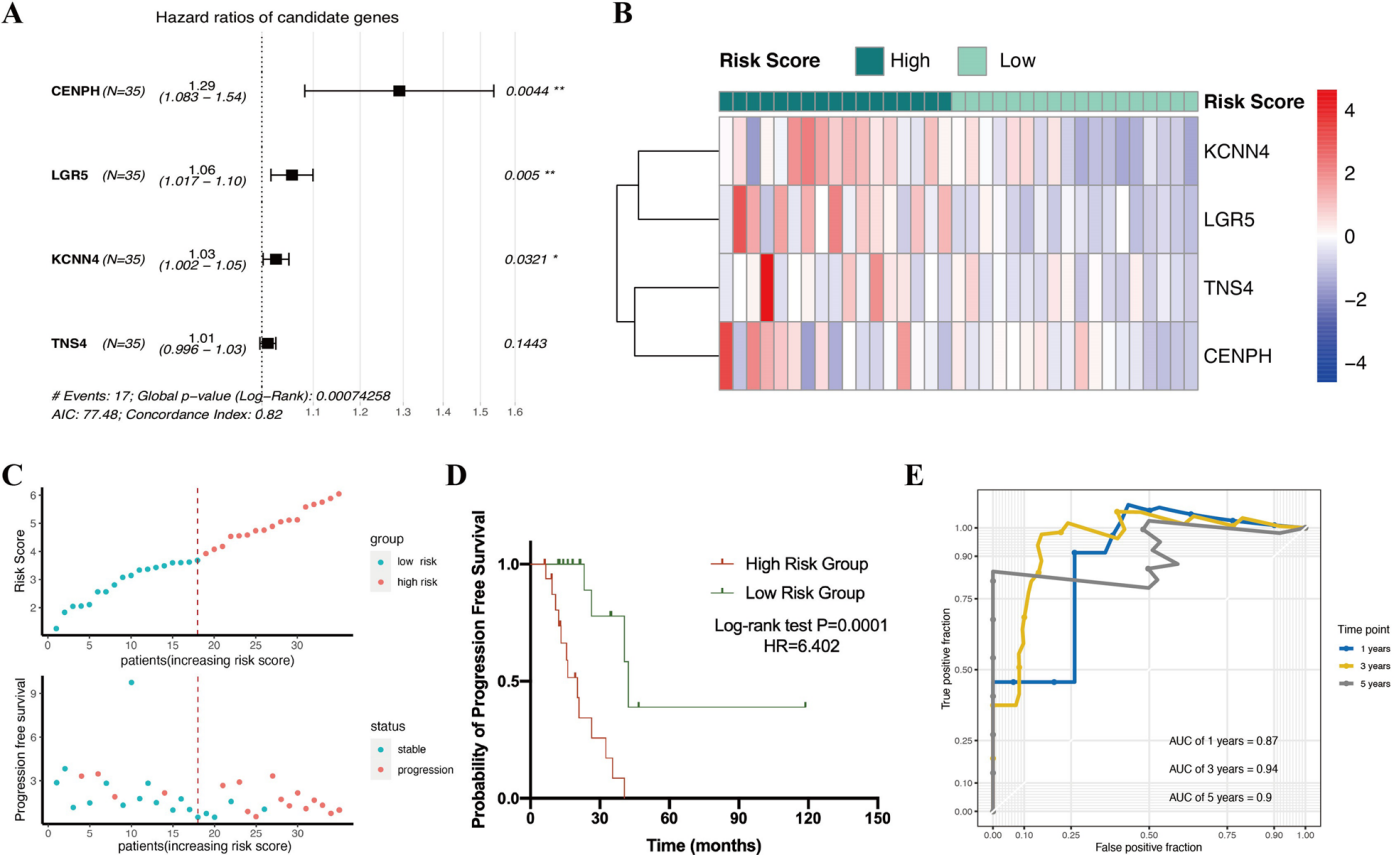
Construction of radiation-resistance gene panel in CO/READ patients to predict prognosis. **a** Forest map of four candidate genes that can predict radiotherapy prognostic. **b** The heat map matrix showed the expression of four genes in 35 enrolled patients who were separated in two risk group by median score. Red-blue represented the level of expression from high to low. **c** Risk score and PFS distribution of enrolled patients. **d** Survival curves of the two groups were drawn based on PFS and follow-up time. **e** ROC curve validated the prognostic efficiency in one, three, and 5 years. ROC, receiver operating characteristic. AUC, area under curve

To investigate the key genes that had the greatest impact on the prognosis of colorectal cancer patients who undergoing radiotherapy, we selected 35 colorectal cancer patients who received radiotherapy in TCGA database to develop univariate and multivariate Cox proportional hazard regression models (baseline information of patients in Additional file 8: Table S3). Among the 252 co-expressed upregulated genes mentioned above, a total of five genes (LGR5, KCNN4, PTRH1, CENPH, TNS4) were considered to be associated with the patients’ PFS in the result of univariate Cox regression model. And among the five genes, LGR5, KCNN4, CENPH, and TNS4 were risk factors. Therefore, we further included those four genes in multivariate regression analysis to construct a gene panel related to radiation resistance. Then, we used coefficients in the multivariate Cox regression and the mRNA expression of key genes to establish a formula to calculate the risk score: Risk score = (CENPH*0.2551 + LGR5*0.05586 + KCNN4*0.02614 + TNS4*0.01099). Hazard ratios superior to 1 indicates that patients with high expression of these four genes are more likely to develop tumor progression after receiving radiotherapy (Fig. 2a). The heatmap clarified the expression level of four genes in each single patient, and all 35 patients were divided into two groups based on the median risk score, with 17 patients in high-risk group and the rest in low-risk group (Fig. 2b, c). As depicted in Fig. 2c, patients with higher risk scores after radiotherapy were more likely to occur tumor progression, and their median progression-free survival were relatively short. Specifically, when we constructed a progression-free survival curve on account of the expression of a single key gene, only one gene could obtain a statistically significant result (KCNN4, P = 0.0042, Additional file 2: Figure S2); but when we grouped patients according to risk score, high-risk group had a significantly higher progression rate in 3 years, 5 years and even longer periods than the low-risk group (P = 0.0001, HR = 6.402) (Fig. 2d), proving the superior performance of this prognostic model. Based on the radiation-resistance gene panel, ROC curve was introduced to reflect this model’s ability in predicting prognosis, with the AUC of 1, 3 and 5- year curves reaching to 0.87, 0.94 and 0.9 respectively, which further indicated a reliable predictive ability (Fig. 2e).

In order to observe the relationship between remaining clinical characteristics and risk score, we established a multivariate regression model (C index = 0.858), including age, gender, stage, T, N, M levels and chemotherapy, which indicated that risk score had a significant association with PFS (P = 0.001) (Additional file 3: Figure S3A). The nomogram was utilized to sketch above mentioned results, combining the risk score and other clinical records, and offered a quantitative tool to predict the probability of progression for each patient (Additional file 3: Figure S3B). What mentioned above suggested the independent prognostic function of risk score (Additional file 9).

### Four genes as the best combination in predicting tumor progression

**Fig. 3:**
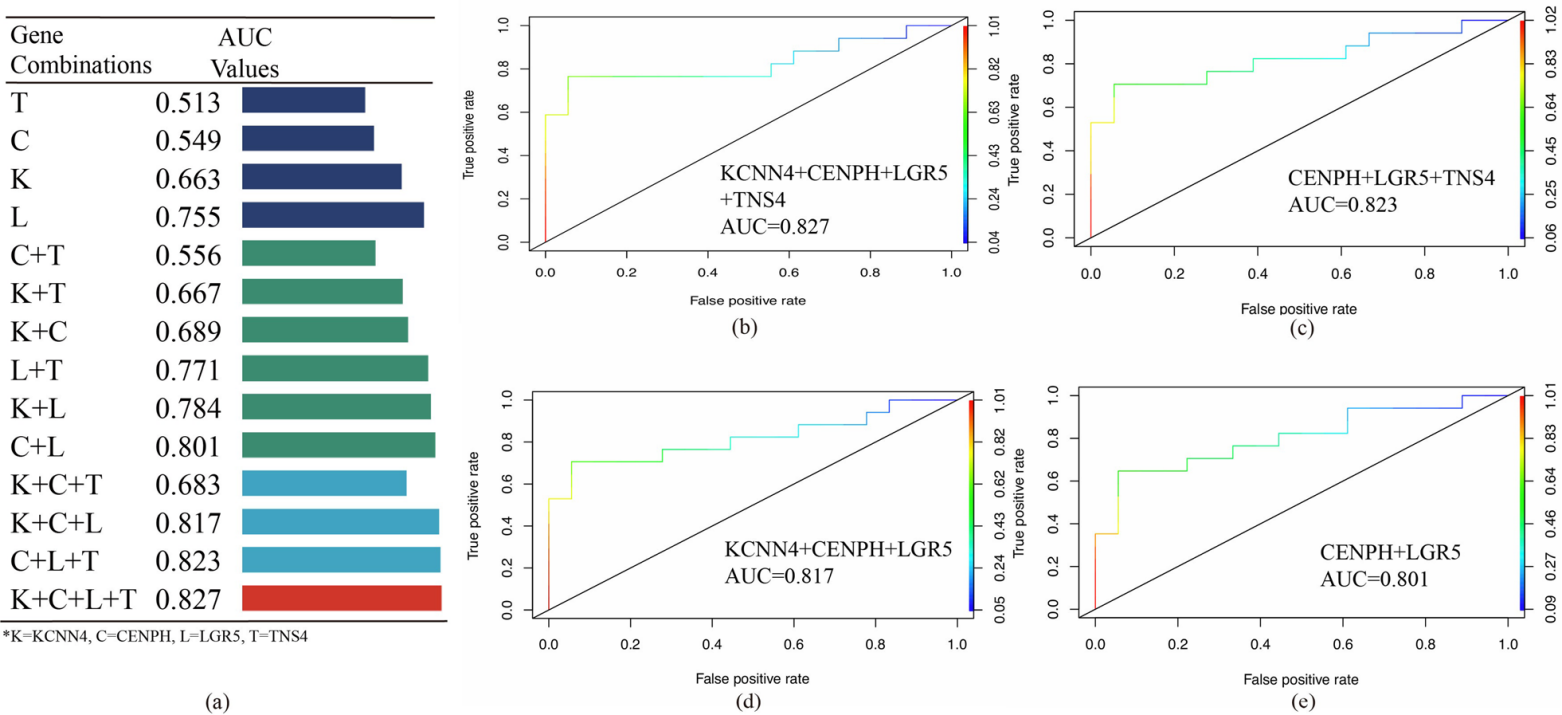
Radiation-resistance gene panel containing four key genes was the best prognostic prediction model. **a** All gene combinations and AUC values corresponding to each one calculated by multivariate logistic regression model. The model built by all four genes had the highest AUC value, representing the best prediction effect. **b**-**d** ROC curves of the best four combinations in predicting clinical outcomes of CO/READ patients after radiotherapy. AUC values were distributed between 0.801 and 0.827

To further verify the reliability of the radiation-resistance gene panel, we established a multivariate logistic regression analysis to evaluate its efficacy to predict tumor progression after radiotherapy. This inspection method can reflect the relationship between gene panel and tumor progression more intuitively. By building a series of regression models with a single gene or combinations with multiple genes, we compared the AUC values in various situations (Fig. 3a). Generally speaking, the AUC values of combinations with two or more key genes were higher than that of a single gene. The AUC value of a single candidate gene ranged from 0.513 for TNS4 to 0.755 for LGR5. Among the various combinations, the one that included all four key biomarkers had the highest AUC value, 0.827, representing the best predictive ability (Fig. 3b). And Fig. 3c–e showed three combinations with AUC values slightly lower than the highest one, reaching 0.823, 0.817 and 0.801 respectively. The data obtained through the above regression models supported our hypothesis that four key genes were participated in radiation resistance of colorectal cancer patients and associated with poor PFS. They may also involve in past-radiotherapy tumor repopulation given the intimate connection between gene panel and PFS.

### Applying WCGNA to link radiation-resistant gene panel and clinical characteristics

After clarifying the reliability of the radiation resistance gene panel in predicting the tumor progression of colorectal cancer patients after radiotherapy, we brought into the weighted gene co-expression network analysis (WGCNA). In this way, we could further explain the correlation between gene panel, PFS and risk score, as well as clustering genes with similar expression patterns to key genes. And the top 10,000 DEGs with the largest variance employed in WGCNA were listed in Additional file 10: Table S5.

**Fig. 4:**
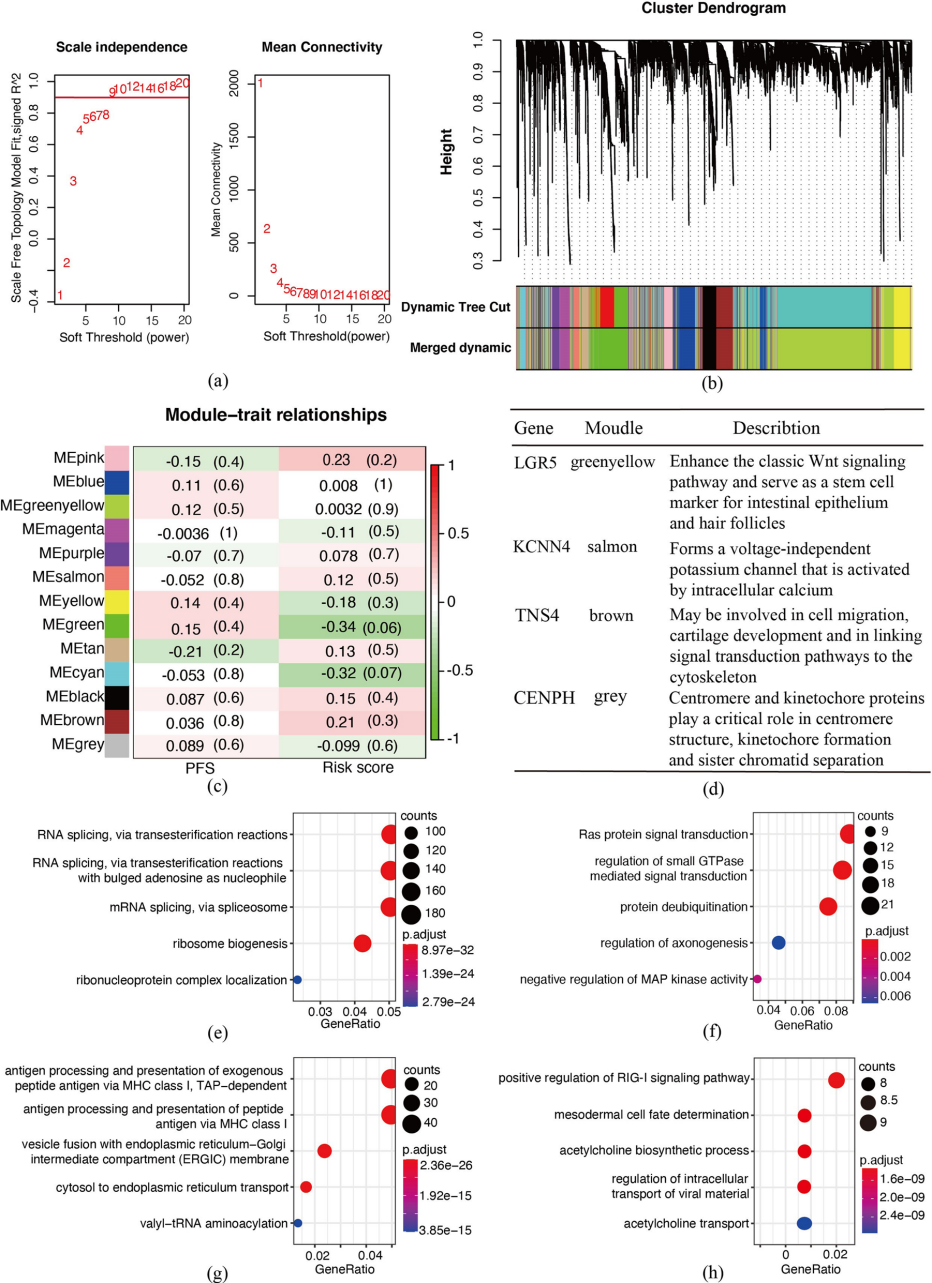
Validating the correlation between clinical trials and radiation resistance gene panel by weighted gene co-expression network. **a** Scale independence and mean connectivity were utilized to pick up the soft threshold of WGCNA (power = 9). **b** The top 10,000 genes of CO/READ patients who received radiotherapy were included to build cluster tree. Similar genes were clustered in gene modules represented by colors (dynamic tree cut), and similar modules were fused to form “merged dynamic”. **c** Heatmap matrix was used to indicate the relationship between gene modules and clinical trials. Gene modules were listed in rows, while PFS and risk score were listed in column. Correlation and P.value were marked in each grid. **d** Gene modules corresponding to the four genes, and a brief overview of gene functions. **e**–**h** Dot chart described pathways enriched by GO analysis. LGR5, KCNN4, TNS4 and CENPH were classified into greenyellow **e**, salmon **f**, brown **g** and grey **h** modules respectively. Gene counts expressed by dot size and P.value by color were listed next to each graph

First, we set the scale free topology model fit R^2 cutoff value to 0.9, and pick beta = 9 as the soft threshold power, building a scale-free network analysis on this basis (Fig. 4a). By merging modules with similar expression profiles in the dynamic tree cut, we eventually got a merged one composed of 13 modules (Fig. 4b). The quantitative connection between each module and clinical trials, PFS and risk score, was shown in Fig. 4c. Generally, PFS and risk score shown a negative correlation trend in post-radiotherapy colorectal cancer patients, providing evidence for the effectiveness of gene panel in forecasting radiation resistance and tumor repopulation. Four key genes in the gene panel were clustered into four different modules respectively (Fig. 4d). Detailed groupings and correlation index (Cor.) were as follows. KCNN4 was clustered in salmon module with 0.119 risk score Cor. and − 0.05 PFS Cor., while TNS4 in brown module with 0.206 risk score Cor. and 0.036 PFS Cor. For these two genes, high risk score corresponded to a relatively negative outcome in PFS. Although LGR5 in green-yellow module (0.032 risk score Cor., 0.115 PFS Cor.) and CENPH in grey module (− 0.099 risk score Cor., 0.089 PFS Cor.) were classified into the low risk score and good PFS outcome modules, the two should be risk factors according to hazard ratios in Cox regression models (Fig. 2a). Moreover, GO enrichment analysis was carried out on the genes in these four modules. And the results were mainly presented as cell cycle control related pathways and activation of immune related pathways caused by the release of intracellular substances in stress status after radiotherapy (Fig. 4e–h).

### *Validating the correlation of radiation-resistance gene PANEL and tumor repopulation in other types of tumors and *in vitro

**Fig. 5:**
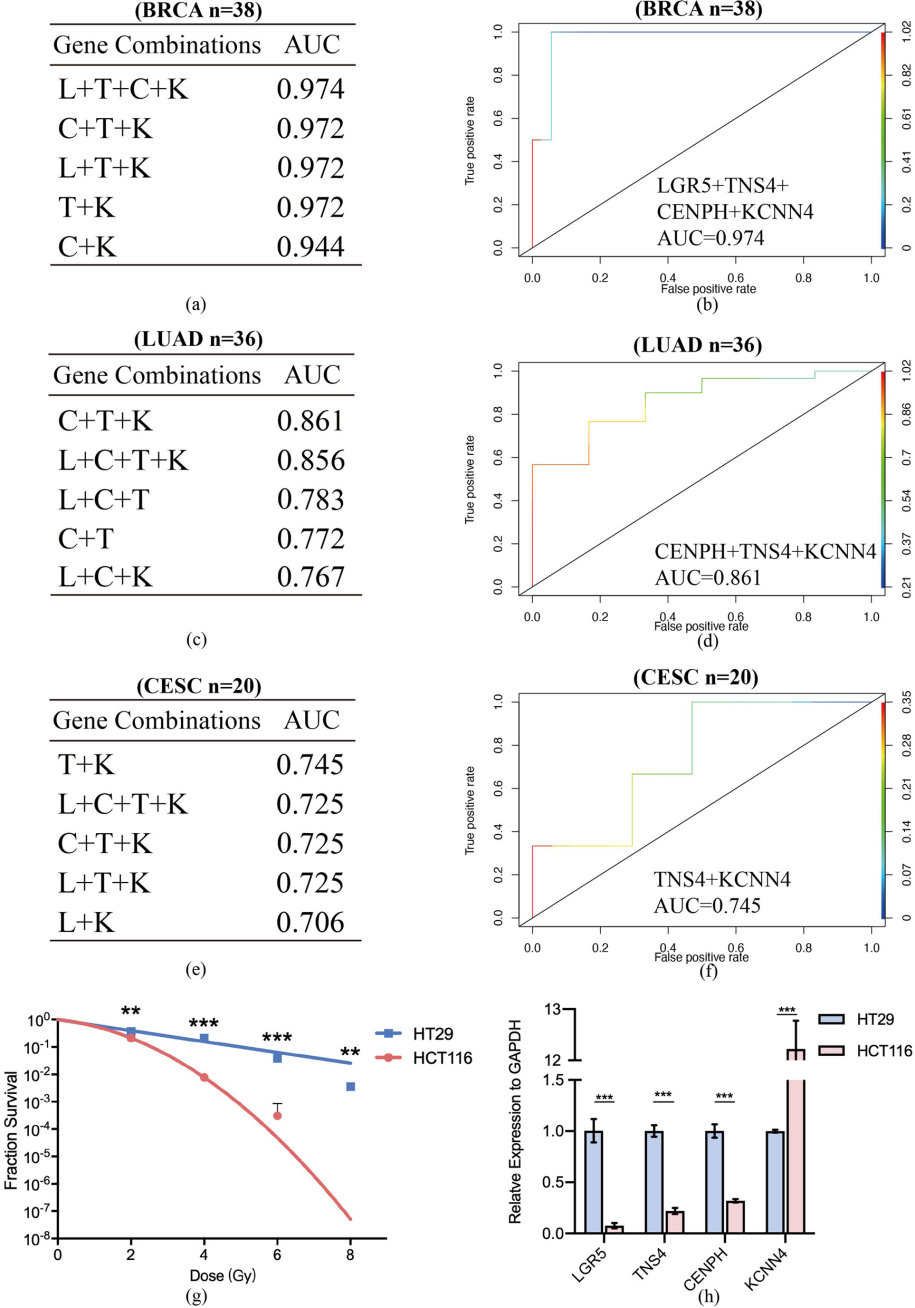
Validating in other TCGA patients with different tumors and colorectal cell lines. **a**, **b** The top five candidate gene combinations with AUC values in TCGA breast cancer patients (**a**), and ROC curve of the best prognostic model (BRCA, n = 38) (**b**). **c**, **d** The top 5 combinations in lung adenocarcinoma patients (**c**) and the best ROC curve (LUAD, n = 36) (**d**). **e**, **f** The top 5 combinations in cervical cancer patients (**e**) and the best ROC curve (CESC, n = 20) (**f**). **g** HT-29 and HCT 116 cells were used for clonogenic formation assay, and the results were displayed through a linear quadratic model. The experiment was repeated three times independently, and statistics were performed by two-tailed ANOVA test. **P ≤ 0.01; ***P ≤ 0.001. **h** Detecting basic mRNA expression of four key genes in two kinds of cells by RT-qPCR. Each sample was repeated three times independently and standardized by GAPDH. ***P ≤ 0.001

In addition to establishing and testing gene panels in colorectal cancer patients, we selected patients in other TCGA projects and used a series of in vitro experiments to further illustrate the sensitivity and specificity of gene panels in predicting radiotherapy resistance and tumor repopulation. Among other types of tumors in the TCGA database, we chose BRCA (n = 38), LUAD (n = 36) and CESC (n = 20) patients who received radiotherapy as the only adjuvant treatment to further proof our hypothesis. Multivariate logistic regression analysis was established in patients of the three kinds of tumor as well, and gene combinations with top 5 AUC values of each cancer were listed in Fig. 5a, c and e. As for breast cancer patients who received radiotherapy, the predictive model containing all four genes reached to the highest AUC value, 0.974 (Fig. 5b). Prognostic model without LGR5 worked best in LUAD patients, with an AUC value of 0.861 (Fig. 5d). In CESC patients, the highest AUC value of 0.745 was a model constructed by KCNN4 and TNS4 (Fig. 5f). In HNSC and ESCA patients, the gene panel also showed acceptable predictive ability (Additional file 4: Figure S4).

Besides, two colorectal cancer cell lines, HT29 and HCT116, were selected for clonogenic formation assay. The results showed that the colony formation ability of HT29 was significantly higher than that of HCT116 after being exposed to various doses of radiation, which meant HT29 cells showed more resistant to radiation (Fig. 5g, Additional file 4: Figure S4). In parallel, the basic expression of radiation-resistance genes in two cells was generally consistent with our expectation, except that KCNN4 had a higher baseline in HCT116 (Fig. 5h).

### Radio-sensitivity of gene knockdown colorectal cell lines

**Fig. 6:**
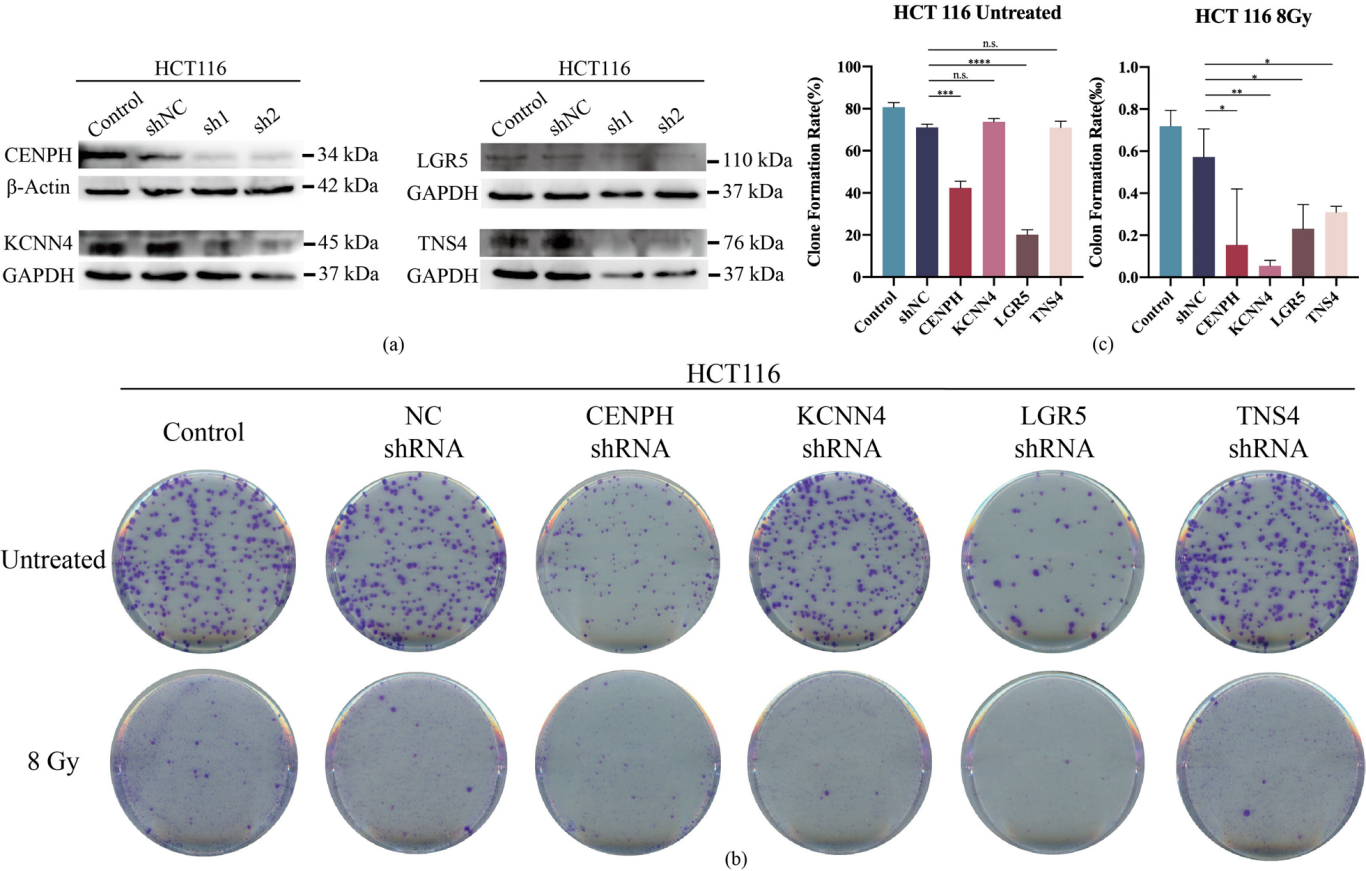
Radio-sensitivity of gene knockdown HCT 116 cell lines. **a** Verification of gene expression in knockdown cells by Western Blot experiments. **b** Clonogenic assay results of untreated group and 8 Gy radiation group. **c** Analysis of Clonogenic assay. n.s. for P > 0.05, * for P ≤ 0.05, ** for P ≤ 0.01, *** for P ≤ 0.001

In order to further confirm the role of the four key genes we found in radio-sensitivity, we constructed four gene knockdown cell lines respectively by using shRNA, and HCT116 cell lines with better knockdown effect were selected through Western Blot experiments (Fig. 6a). Then, we carried out clone formation assay with these cells (Fig. 6b). Compared with the control cells transfected with empty plasmid (NC shRNA), a decreased clonogenic ability of CENPH and LGR5 knockdown cells could be observed in the untreated group, while the ability of KCNN4 and TNS4 knockdown cells was not significantly affected (Fig. 6c). However, when treated after 8 Gy irradiation, CENPH knockdown cells could hardly form clone, and the other three gene knockdown cells could only form few clones (Fig. 6b). And the clonogenic ability of all knockdown cells decreased unexpectedly after radiation treatment compared with the control groups (Fig. 6c). The same gene knockdown and clonogenic assay for further validation were also carried out in Ht29 cells (Additional file 5: Figure S5).

## Discussion

Resistance to therapy, which contributes a lot to poor prognosis of cancer patients, is an unavoidable part of cancer treatment, and this situation is most common in patients with recurrence and metastasis. Targeted therapy and chemotherapy are the hardest hit areas for therapy resistance and tumor relapse [25–27]. It is ordinary for tumor with a strong initial response to eventually develop into a drug resistant one [25]. Under the stimulation of treatment, drug-resistance tumor cells that successfully escape cytotoxicity become the dominant group in tumor repopulation [3, 6, 25]. Traditional theories believed that this group of cells acted as cancer stem cells (CSCs) to mediate tumor relapse through genetic processes [28, 29], while recent research results used persistent state or slow cycling state, a diapause-like slow proliferation state that mediates relapse through non-genetic mechanisms, to describe this group of cells [7, 8, 10, 30]. Similarly, some primary tumors that were originally sensitive to radiotherapy show resistance behavior after recurrence in clinical practice, indicating that radiation resistant tumor cells are the culprit of tumor repopulation after radiotherapy. Therefore, our research focused on the role of radiation-resistance cells in tumor repopulation after radiotherapy (Additional file 11).

To explore this question, our attention focused on radiation resistant colorectal cancer cells, which were the consequent of repeated induction by radiation. In our previous research, we screened the targeted therapy dataset of lung cancer to predict the efficacy of radiotherapy [31]. In this study, we used a radiation related dataset and conducted more complete experimental verification. Through the integration and differential analysis of sequencing data from radiation resistant colorectal cancer cells as well as TCGA tumor and normal tissue data of colorectal cancer patients, the co-upregulated genes were spotted. While representing increased resistance to radiotherapy, this group of genes also represented a stronger tumor proliferation capability. Based on the TCGA training set of 35 colorectal cancer patients undergoing radiotherapy, we developed a gene panel containing four genes by condensing these co-upregulated genes. And predictive ability of the gene panel was validated through five different datasets and in vitro experiments, to make the verification process more convincing.

In this work, GO analysis of the obtained 710 upregulated genes in resistant cells clarified that cell cycle, DNA replication and repair pathways were significantly enriched, which indicated that resistant cells were timely and effective in dealing with DNA damage than sensitive cells (Fig. 1b). But whether faster recovery from DNA damage response meant stronger proliferative capacity was largely unknown. Therefore, we included the TCGA-COREAD database (tumor vs. normal tissues) that could reflect tumor proliferation ability into the analysis, and found a total of 252 co-upregulated genes intersecting with GEO dataset, implying the tendency of radiation-resistance cells to participate in tumor repopulation (Fig. 1d). Enrichment analysis of these co-upregulated genes showed that pathways related to cytokinesis were activated (Additional file 1: Figure S1). This is consistent with previous studies on radiation resistant cells, which hold the view that cancer stem cells were more resistant to radiation, especially manifesting in DNA damage repair and ROS scavenging capabilities, and their stemness characteristics were closely related to accelerated regeneration during or after treatment [24, 32, 33].

In order to further target the radiation-resistance genes closely related to tumor repopulation after radiotherapy, we used multivariate regression analysis to condense 252 co-upregulated genes and established a prognostic prediction system, which consisted of four key genes (LGR5, KCNN4, TNS4, CENPH), based on the TCGA colorectal cancer patients (Figs. 2 and 3). Meanwhile, four high-risk genes related to bad PFS were verified their prognostic ability in BRCA, LUAD, CESC, ESCA and HNSC patients (Fig. 5 and Additional file 4: Figure S4).

LGR5 (Leucine-rich repeat containing G protein-coupled receptor), which is regarded as a marker of adult stem cells, is a gene encoding for a composition of the Wnt receptor complex [34, 35]. After activation, LGR5 recruits the LRP receptor complex which can bind to Wnt ligand [36]. And β-catenin is then further accumulated and transported to the nucleus binding with TCF/LEF family of transcription factors, which induce the expression of Wnt target genes, including C-myc and cyclinD1, stimulating tumor cell proliferation, EMT and other processes [36]. These are confirmed in many studies. A recent study revealed that LGR5 + colon CSCs were responsible for driving tumor re-growth after ablation [37]. And LGR5 knockdown reduced tumor invasion and migration and blocked EMT by inhibiting the Wnt/β-catenin pathway, in both breast cancer and glioma [38, 39]. In another study about 5-Fu induced drug-resistant colorectal cancer cells, authors found that LGR5 + tumor cells were significantly enriched in pool of resistant cells by constructing an organoid model, which was similar to our analysis that LGR5 was highly expressed in radio-resistant cells [40].

KCNN4, a potassium channel protein activated by Ca2 + , is implicated in the promotion of cell invasion and cell proliferation, and has been considered as a poor prognostic factor for thyroid cancer [41], pancreatic cancer [42, 43], lung cancer [44] and glioblastoma [45]. AP-1, as a transcription factor induced by various stress, promotes the overexpression of KCNN4, which may depend on the Ca2 + /MET/AKT axis to exert its function [46]. It was reported that KCNN4 regulated calcium ion signals to influence the cell cycle arrest and promote the repair of damaged DNA in glioma, thereby increasing the radio-resistance of tumors [45, 47]. What’s more, evidence also shown that KCNN4 was upregulated by PRL-3 to promote the proliferation of colorectal cancer cells, and contributed to the invasion and metastasis of colorectal cancer by participating in the PRL-3 mediated EMT process [48, 49]. Various evidence indicates that KCNN4 is an important factor in radiation resistance and tumor proliferation.

TNS4 is a focal adhesion molecule that belongs to the tensin family, and it is significantly up-regulated in a variety of gastrointestinal tumors and lung cancer [50–52]. It regulated cell survival, proliferation, and migration through increased MET protein stability in colorectal cancer [53]. Moreover, TNS4 expression was significantly increased in hepatocellular carcinoma and intrahepatic cholangiocarcinoma and was positively feedback-regulated by KRAS and SOX17 to stimulate migration and proliferation [54, 55]. In addition, TNS4 could inhibit the degradation of EGFR, a molecule related to tumor cell proliferation and apoptosis inhibition, through post-translational modification, and prolonged its function [56]. These are in agreement with the higher survival and proliferation capacity of radiation-resistant cells in our research.

As one of the essential components of active kinetochore, overexpression of centromere protein H (CENPH) in human colorectal cancer was shown to be a major cause of chromosomal instability (CIN) [57]. This state is an important boost in driving tumor cells to produce anti-therapeutic mutations and promoting tumor evolution [58, 59]. And CENPH has been considered to be associated with tumor progression and poor prognosis in NSCLC [60], tongue cancer [61], esophageal cancer [62] and gastric cancer [63]. And the effect of CENPH may be highly related to Survivin, an inhibitor of apoptosis protein family, which helps tumor cells to survive and restore proliferation under harmful stress [61, 63]. Meanwhile, knockdown of CENPH retarded the growth of Hep3B, hepatic carcinoma cell, subcutaneous xenograft, and decreased the expression of Ki-67 and BCL-2 [64]. High expression of this gene in radiation-resistant cells probably indicates enhanced proliferation capability. A brief description of four key genes was shown in Fig. 4d.

There are still some limitations that need to be further improved in this study. Firstly, the gene panel needs to be validated in patient cohorts. The validation part of this study relies on TCGA clinical data and in vitro experiments, which cannot fully represent the complexity and heterogeneity of human tumors. Secondly, the possible mechanisms involved in key genes are needed to be clarified. Research on mechanisms may throw light on these key genes to become therapeutic targets. To make up for these shortcomings in the future study, we hope to further include large sample size and well characterized clinical patient cohorts to test the ability of the gene panel in predicting the prognosis of colorectal cancer patients receiving radiotherapy. Through real-world clinical research, we will gain a more specific understanding of how the obtained gene panel will guide treatment decisions. And through sequencing technology, tumor biopsy from patients can also help us understand mechanisms of radiation resistance and tumor repopulation.

## Conclusions

In conclusion, through the sorting and analysis of the radiation resistance projects in the GEO database and tumor vs. normal tissue project in TCGA-COREAD database, we established a gene panel (LGR5, KCNN4, TNS4 and CENPH) in colorectal cancer patients receiving radiotherapy. It reflected the relationship between radiation- resistance and tumor repopulation, and was verified to be an acceptable indicator of prognosis for colorectal cancer. These molecules may become new targets to predicate tumor repopulation after radiotherapy for colorectal cancer patients.

## Supplementary Information

**Additional file 1: Fig. S1.** **A** GO analysis and **B** chord diagrams of 252 co-upregulated genes.

**Additional file 2: Fig. S2.** Survival curves of each single gene. Patients were grouped by median mRNA expression of each gene.

**Additional file 3: Fig. S3.** **A** Multivariate regression analysis combined with clinical information, and **B** a nomogram reflecting the ability to predict progress.

**Additional file 4: Fig. S4.** Representative evidence of further validation in TCGA and clone formation assay. **A**, **B** The top five candidate gene combinations with AUC values in TCGA head and neck cancer (n=95) and esophageal cancer (n = 17), and ROC curve of the best prognostic model. **C** Using HCT116 and HT29 cells treated by various radiation dose. **D** Statistics of the number of clones formed. The experiment was repeated three times for each dose. **E** Clone formation rate of the two cells.

**Additional file 5: Fig. S5.** Radio-sensitivity of gene knockdown Ht29 cell lines. **A** Verification of gene expression in knockdown cells by Western Blot experiments. **B** Clone formation assay results of 8Gy radiation group and untreated group. **C** Analysis of clone formation assay.

**Additional file 6: Table S1.** DEGs of dataset GSE97543.

**Additional file 7: Table S2.** DEGs of dataset TGCA-CO/READ.

**Additional file 8: Table S3.** Baseline of cancer patients in the training set and validating set.

**Additional file 9: Table S4.** Candidate genes obtained by univariate Cox regression analysis.

**Additional file 10: Table S5.** The top 10,000 DEGs employed in WGCNA.

**Additional file 11: Table S6.** Primer for RT-qPCR, shRNA sequences and antibody information.

## Data Availability

We enrolled public datasets to construct this research. They can be obtained in Gene Expression Ominibus (GEO, https://www.ncbi.nlm.nih.gov/geo/), GSE97543; and The Cancer Genome Atlas (TCGA, https://portal.gdc.cancer.gov/), program: TCGA-COAD, TCGA-READ.

## Abbreviations

DEGs: Differential expression genes
GEO: Gene Expression Ominibus
TCGA: The Cancer Genome Atlas
CO/READ: Colon and rectal adenocarcinoma
ROC: Receiver operating character
AUC: Area under the curve
BRCA: Breast carcinoma
LUAD: Lung adenocarcinoma
CESC: Cervical squamous cell carcinoma and endocervical adenocarcinoma
HNSC: Head and neck squamous cell carcinoma
ESCA: Esophageal carcinoma
