# Supplementary figures and images for: Predicting tumor repopulation through the gene panel derived from radiation resistant colorectal cancer cells
Source: J Transl Med. 2023 Jun 16;21:390. doi: 10.1186/s12967-023-04260-x (PMC10273655; doi:10.1186/s12967-023-04260-x)

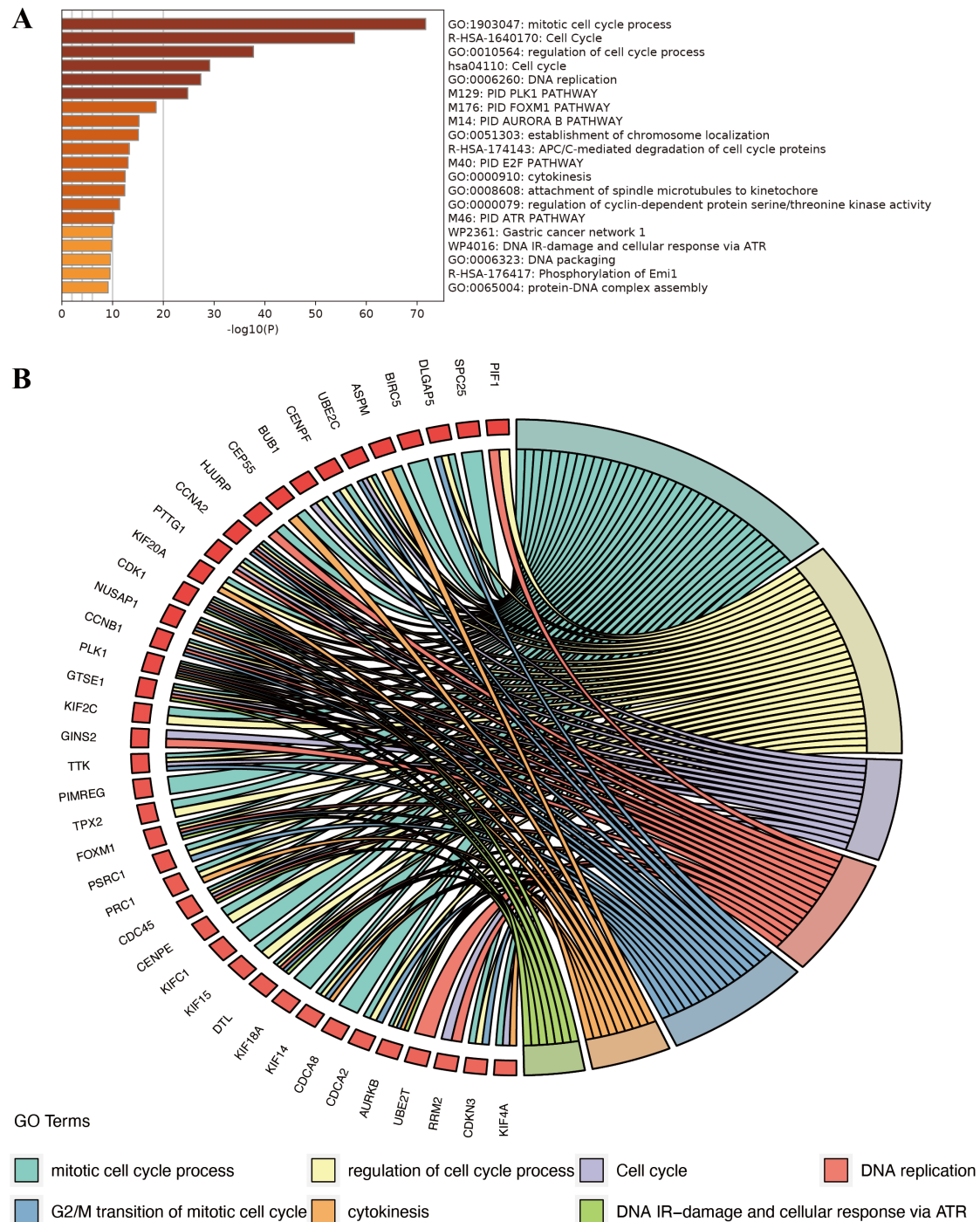

**Supplementary Figure 1.** GO analysis **(A)** and chord diagrams **(B)** of 252 co-upregulated genes.

Supplement: Supplementary file 1 — Additional file 1: Fig. S1. A GO analysis and B chord diagrams of 252 co-upregulated genes. [file 12967_2023_4260_MOESM1_ESM.pdf]
