# Supplementary material for: Predicting tumor repopulation through the gene panel derived from radiation resistant colorectal cancer cells
Source: J Transl Med. 2023 Jun 16;21:390. doi: 10.1186/s12967-023-04260-x (PMC10273655; doi:10.1186/s12967-023-04260-x)
Supplement: Supplementary file 2 — Additional file 2: Fig. S2. Survival curves of each single gene. Patients were grouped by median mRNA expression of each gene. [file 12967_2023_4260_MOESM2_ESM.pdf]

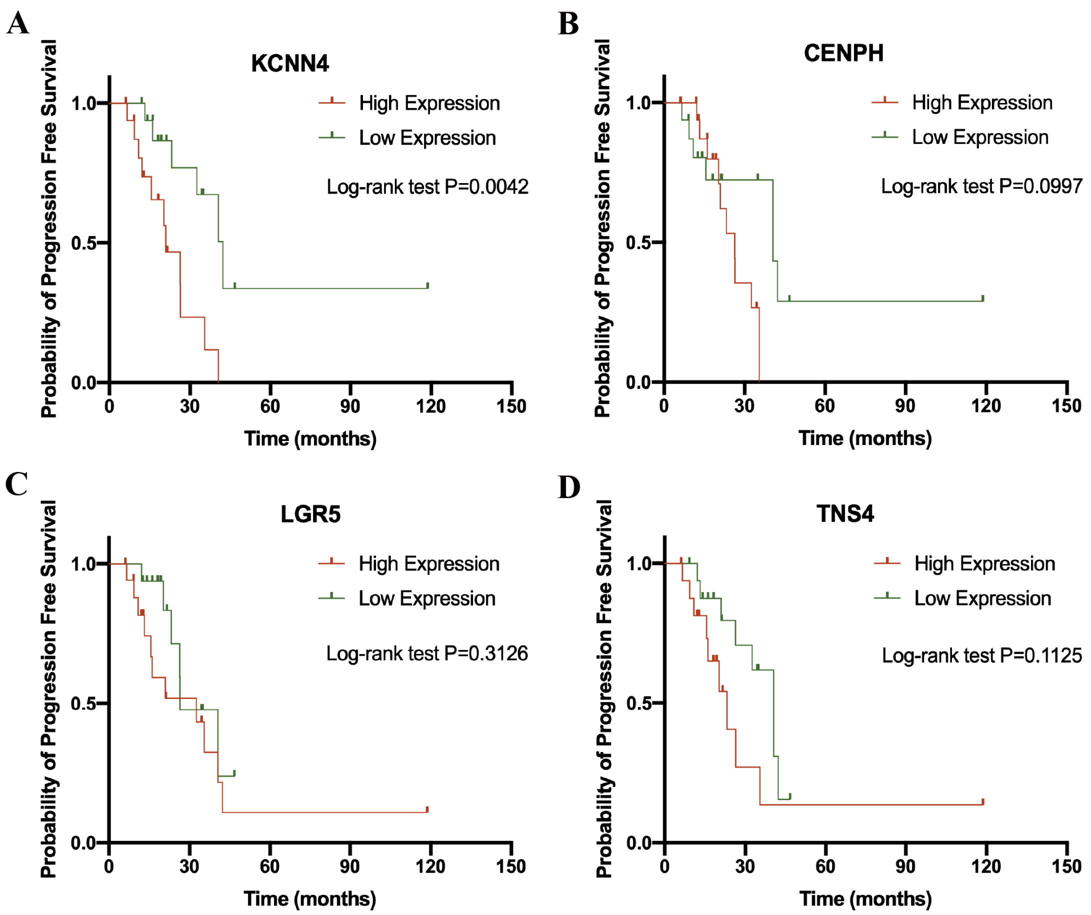

Supplementary Figure 2. Survival curves of each single gene. Patients were grouped by median mRNA expression of each gene.
