# Supplementary material for: Predicting tumor repopulation through the gene panel derived from radiation resistant colorectal cancer cells
Source: J Transl Med. 2023 Jun 16;21:390. doi: 10.1186/s12967-023-04260-x (PMC10273655; doi:10.1186/s12967-023-04260-x)
Supplement: Supplementary file 3 — Additional file 3: Fig. S3. A Multivariate regression analysis combined with clinical information, and B a nomogram reflecting the ability to predict progress. [file 12967_2023_4260_MOESM3_ESM.pdf]

A

Characteristics	Hazard.Ratio	CI95	P. Value
Age	1.01	0.94-1.07	0.861
Stage (I/II/III/IV)	0.34	0.03-4.01	0.393
Gender (Male/Female)	2.02	0.49-8.34	0.329
T Stage (1/2/3/4)	2.66	0.19-37.75	0.471
N Stage (0/1/2)	2.45	0.59-10.19	0.218
M Stage (0/1)	5.92	0.26-136.14	0.266
Chemotherapy	0.63	0.14-2.80	0.541
Riskscore	2.82	1.57-5.06	0.001

B

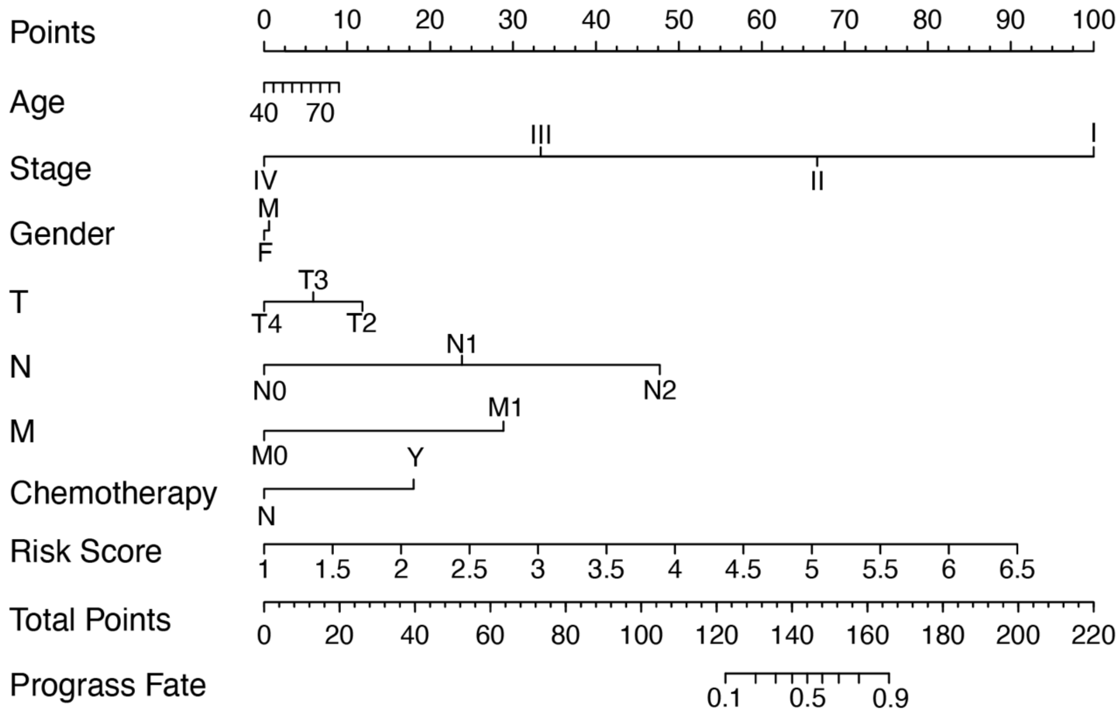

Supplementary Figure 3. Multivariate regression analysis combined with clinical information **(A)**, and a nomogram reflecting the ability to predict progress **(B)**.
