# Supplementary material for: Predicting tumor repopulation through the gene panel derived from radiation resistant colorectal cancer cells
Source: J Transl Med. 2023 Jun 16;21:390. doi: 10.1186/s12967-023-04260-x (PMC10273655; doi:10.1186/s12967-023-04260-x)
Supplement: Supplementary file 4 — Additional file 4: Fig. S4. Representative evidence of further validation in TCGA and clone formation assay. A, B The top five candidate gene combinations with AUC values in TCGA head and neck cancer (n=95) and esophageal cancer (n = 17), and ROC curve of the best prognostic model. C Using HCT116 and HT29 cells treated by various radiation dose. D Statistics of the number of clones formed. The experiment was repeated three times for each dose. E Clone formation rate of the two cells. [file 12967_2023_4260_MOESM4_ESM.pdf]

A

(HNSC n=95)

Gene Combinations	AUC
C+L+K	0.706
T+L+K	0.703
C+L+T+K	0.701
C+T+K	0.670
K+T	0.657

(a)

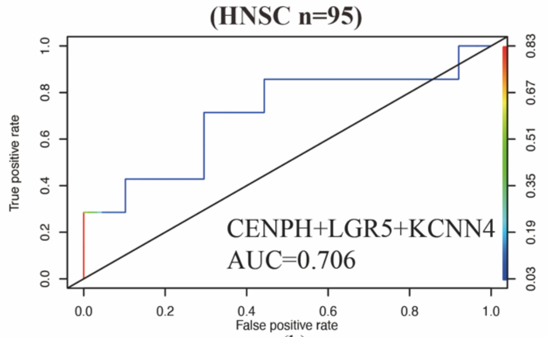

(b)

B

(ESCA n=17)

Gene Combinations	AUC
C+L+K	0.697
C+L+T+K	0.682
L+T+K	0.667
C+T+K	0.636
T+K	0.606

(a)

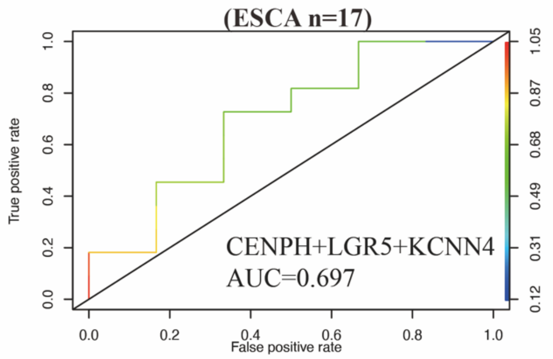

(b)

C

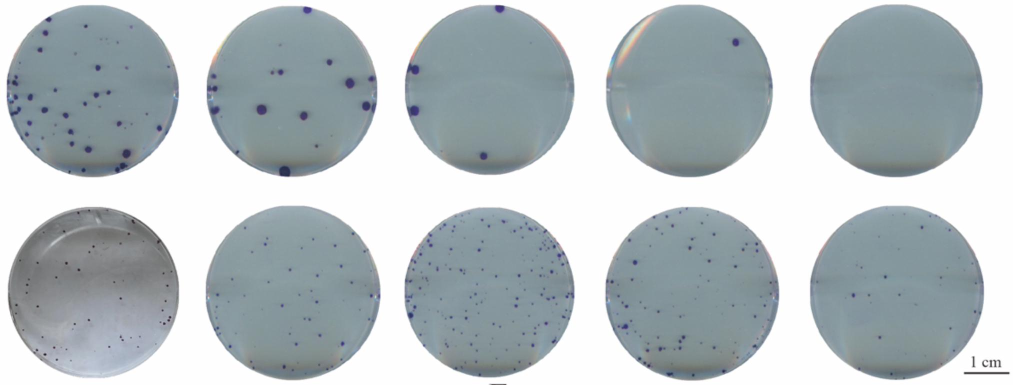

D

	HCT116			HT29		
NC (100 cells/well)	42	47	40	48	52	50
2Gy (200 cells/well)	20	19	16	32	42	37
4Gy (1000 cells/well)	4	3	3	86	119	108
6Gy (2500 cells/well)	1	0	0	54	52	39
8Gy (10000 cells/well)	0	0	0	13	21	19

E

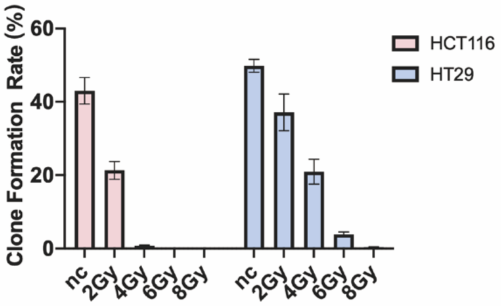

Supplementary Figure 4. Representative evidence of further validation in TCGA and clonogenic formation assay. (A) (B) The top five candidate gene combinations with AUC values in TCGA head and neck cancer (n=95) and esophageal cancer (n=17), and ROC curve of the best prognostic model. (C) Using HCT116 and HT29 cells treated by various radiation dose. (D) Statistics of the number of clones formed. The experiment was repeated three times for each dose. (E) Clone formation rate of the two cells.
