# Supplementary material for: Predicting tumor repopulation through the gene panel derived from radiation resistant colorectal cancer cells
Source: J Transl Med. 2023 Jun 16;21:390. doi: 10.1186/s12967-023-04260-x (PMC10273655; doi:10.1186/s12967-023-04260-x)
Supplement: Supplementary file 5 — Additional file 5: Fig. S5. Radio-sensitivity of gene knockdown Ht29 cell lines. A Verification of gene expression in knockdown cells by Western Blot experiments. B Clone formation assay results of 8Gy radiation group and untreated group. C Analysis of clone formation assay. [file 12967_2023_4260_MOESM5_ESM.pdf]

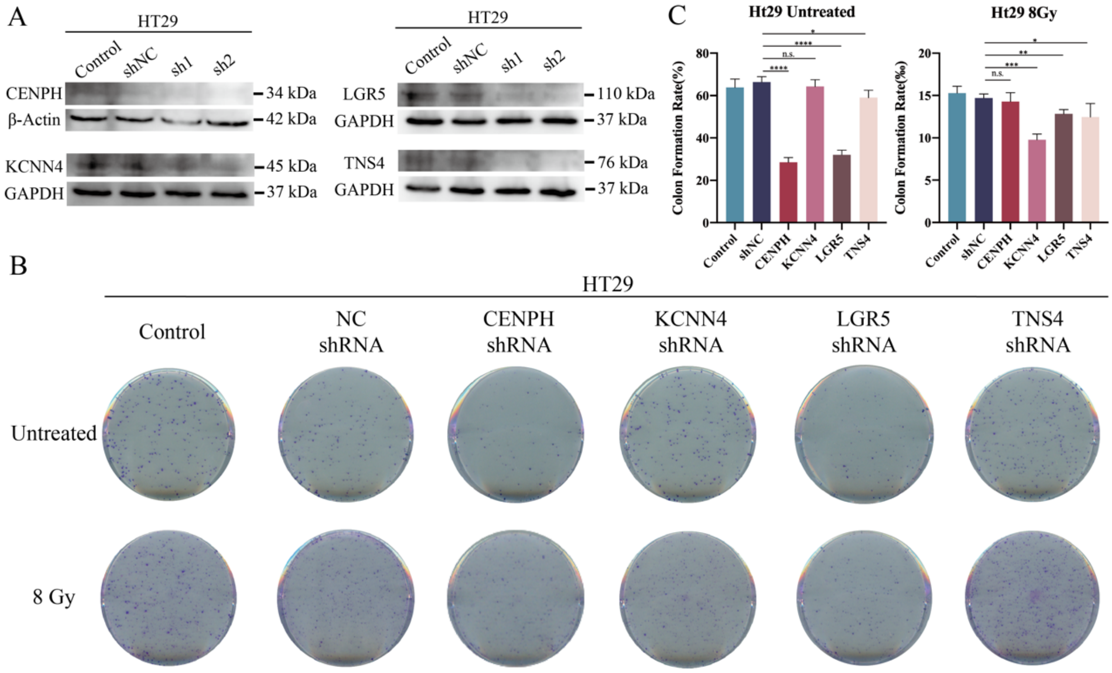

Supplementary Figure 5. Radio-sensitivity of gene knockdown Ht29 cell lines. (A) Verification of gene expression in knockdown cells by Western Blot experiments. (B) Clone formation assay results of 8Gy radiation group and untreated group. (C) Analysis of clone formation assay.
