# Supplementary material for: Predicting tumor repopulation through the gene panel derived from radiation resistant colorectal cancer cells
Source: J Transl Med. 2023 Jun 16;21:390. doi: 10.1186/s12967-023-04260-x (PMC10273655; doi:10.1186/s12967-023-04260-x)
Supplement: Supplementary file 11 — Additional file 11: Table S6. Primer for RT-qPCR, shRNA sequences and antibody information. [file 12967_2023_4260_MOESM11_ESM.docx]

Table S6:

The qRT-PCR primers employed are as follows:

| Gene | Forward (5’-3’) | Reverse (5’-3’) |
| --- | --- | --- |
| LGR5 | CACCTCCTACCTAGACCTCAGT | CGCAAGACGTAACTCCTCCAG |
| KCNN4 | TTGGCTGATCCCCATCACATT | CAGGCTTCTTGTAGCACTCGG |
| CENPH | GCTGAGAGCACAGACAAAACA | GCAGTTGAAAGTCTCATCCTGTCT |
| TNS4 | GCTATATGTTTGGAAGCAGCCAG | TTAGGGTGGGGTTTCTGGGA |
| GAPDH | CCGGGAAACTGTGGCGTGATGG | AGGTGGAGGAGTGGGTGTCGCTGTT |

The shRNA employed are as follows:

| Gene | Sh1 (5’-3’) | Sh2 (5’-3’) |
| --- | --- | --- |
| LGR5 | CCATCCAATTTGTTGGGAGAT | CCATAGCAGTTCTGGCACTTA |
| KCNN4 | GCCTGGATGTTCTACAAACAT | CATGATGGATATCCAGTATAC |
| CENPH | GAGACTTTCAACTGCACTTAA | GCTTGAGAAGAATGTTGACAT |
| TNS4 | CAATGACCTCATCCGACACTT | GAAGTGGCAGAAGTACTGCAA |

Antibody information:

| Gene | Product No. | Dilution ratio |
| --- | --- | --- |
| LGR5 | ABclonal A12327 | 1:1000 |
| KCNN4 | Santa Cruz sc-365265 | 1:500 |
| CENPH | Santa Cruz sc-365222 | 1:500 |
| TNS4 | Proteintech 11580-1-AP | 1:1000 |
